# The Severity of Interstitial Inflammation in the Renal Parenchyma of Albino Rats Is Subjected to the Dose of Heavy Metals

**DOI:** 10.7759/cureus.25307

**Published:** 2022-05-24

**Authors:** Hira Kareem, Amna Jahan, Rajia Liaqat, Tahira Liaqat, Sobia Jahangir, Hamza Shahab

**Affiliations:** 1 Department of Pathology, Services Institute of Medical Sciences/Services Hospital, Lahore, PAK; 2 Department of Histopathology, Services Institute of Medical Sciences, Lahore, PAK; 3 Department of Pathology, Al-Aleem Medical College, Lahore, PAK; 4 Department of Pathology, Services Institute of Medical Sciences, Lahore, PAK; 5 Department of Physics, University of Wah, Rawalpindi, PAK

**Keywords:** morphology, interstitium, inflammation, fibrosis, edema, copper

## Abstract

Aim

This study aimed to determine the relationship between irreversible morphological changes in the renal interstitium and the duration of exposure to heavy metals such as copper in albino rats.

Materials and methods

An experimental research design was used to conduct this study from November 2019 to May 2020. All experiments were performed in the Department of Pathology of the Services Institute of Medical Sciences, University of Health Sciences (UHS) (animal house), Lahore, Pakistan. A total of 30 albino rats equally divided into three groups were included in the study. Group I (control) was given tap water and a typical rodent pellet diet. Groups II and III (experimental) were fed with copper (heavy metal) at a dose of 0.15 and 0.30 mg/kg body weight, respectively, for 18 weeks on alternate days. At the end of the experiment, the kidneys were extracted from the rats, stained with hematoxylin and eosin, and processed for histological observation. Renal histopathological changes were evaluated in terms of edema, inflammation, and fibrosis.

Results

The collected data were analyzed using the Chi-square test, with p < 0.05 considered significant. Renal histopathology in terms of interstitium showed that edema, inflammation, and fibrosis were significantly different in all groups. In Group I, none of the rats had edema, inflammation, and fibrosis, while in Groups II and III, these characteristics were observed; the difference was significant between the experimental and control groups.

Conclusions

Heavy metals, such as copper, can induce renal parenchymal changes in a dose-dependent manner, resulting in edema, fibrosis, and inflammation.

## Introduction

The kidneys are complex and sophisticated organs. Metabolic wastes are excreted through the kidneys via a specific circulatory function, which has the capacity to regulate hemodynamic responses [1].

Heavy metals that are abundant in the environment and are hazardous to the renal tubules are deposited in the kidneys. These heavy metals are trapped inside the Bowman’s capsule, which then induces inflammatory responses. Heavy metals, such as copper, can cause renal tissue injury that may lead to further damage to the proximal convoluted tubules. Extra composites of heavy metals lead to denudation of the tube-shaped cells and mobbing of the glomeruli [2]. The renal medulla then becomes edematous, spots develop on the tubular epithelium, and eosinophilic-type casts form inside the interstitium due to the deposition of excessive amounts of copper [3]. Other changes include capillary dilatation of glomerulus, mobbed kidneys, and interstitial penetration of lymphocytes [4,5].

Several factors also influence the level of heavy metal toxicity, such as the absorbed amount, heavy metal toxicity, exposed individual’s age, and exposure route [6]. As some heavy metals are used for a prolonged period to treat certain conditions, they can cause permanent renal damage, which may ultimately result in death [7]. To our knowledge, previous reporting of the histochemical alterations in the renal tissues due to copper exposure is limited and has not yet been well identified in Pakistan. Thus, in this study, we hypothesized and attempted to investigate the relationship between irreversible morphological changes in the renal interstitium and the duration of exposure to copper in albino rats.

## Materials and methods

Study design

This randomized controlled study used adult Wistar albino rodents [8]. Institutional review board approval was granted by the Services Institute of Medical Sciences, Lahore, Pakistan (REF/SIMS/181U). According to the extant literature, the experiment period was 18 weeks [9]. The laboratory work was conducted in the Department of Pathology of the Services Institute of Medical Sciences from December 3, 2019, to April 17, 2020, and the animals were kept in the animal house of the University of Health Sciences (UHS), Lahore.

Experimental animal

Wistar albino rodents were considered suitable samples to study the effects of copper on the kidneys [9]. Thirty rodents, aged six to eight weeks and weighing between 200 and 250 grams, were used for the study [10]. The rodents were kept in separate cages with light-dark cycles of 12 hours each, temperatures between 22°C and 25°C, and humidity of 65% ± 5%. The prescribed dose of copper for each rodent was administered on alternate days.

Experimental design

The 30 rodents were divided into three groups of 10 rats per group and were treated as follows. In Group I, 10 healthy rodents were given distilled water along with a normal diet for 18 weeks. In Group II, 10 healthy rodents received tap water along with oral food on alternate days for 18 weeks. The food was given in the form of pellets with copper at a dose of 0.15 mg/kg body weight, homogenized, and mixed with wheat flour. In Group III, 10 healthy rodents received tap water along with oral food on alternate days for 18 weeks. The food was given in the form of pellets with copper at a dose of 0.30 mg/kg body weight, homogenized, and mixed with wheat flour.

Diet of Wistar albino rodents

The exact quantification of copper mixed with the food of Wistar albino rodents was based on the guide from the Biochemistry Department of UHS. According to their suggestion, the rodents of Group II were given the customized pellets.

Assessment of the oral LD50

The oral lethal dose (LD50) of copper in rats was reported to be 2 mg/kg body weight, similar to that in the literature [11]. This dose was adjusted daily according to the weight of each rodent.

Sample collection for histopathology

At the end of the experiment, the animals were sacrificed under anesthesia and dissected according to proper procedures and ethics. The kidneys were extracted and examined both macroscopically and microscopically [12].

Histopathological examination

For histopathological analysis, a fixative having absolute alcohol (60 mL), glacial acetic acid (10 mL), and formaldehyde (30 mL) were utilized to fix the renal tissues. For the preparation of slides, 5 µm thin sections of fresh kidney tissues were used. For staining purposes, the hematoxylin and eosin stain was used, and images were taken at a magnification of 40× under a compound microscope for histopathological study. The kidneys were also processed for Jones’ methenamine silver and periodic acid-Schiff stains. Microscopy was done, and it was estimated whether edema, inflammation, and fibrosis were present or not. A quantitative scoring system was used to assess histopathological alterations according to the method illustrated by Toya et al. [13] with some modifications, and the data was filled in relevant proformas.

Statistical analysis

Data were entered and analyzed using SPSS version 20.0 (IBM Corp., Armonk, NY, USA). Frequencies and percentages were given for qualitative variables such as histopathological changes in kidneys, which were analyzed using Chi-square tests. A p-value of ≤0.05 or equivalent was considered statistically significant.

## Results

Glomerular changes

Histopathological examination in terms of glomeruli shows that the mesangium and capillary wall of the rats in Group I were normal, while in Groups II and III, the mesangium was expanded, and the capillary wall was thickened in all rats. Necrosis, thrombosis, fibrin deposits, crescent formation, and sclerosis were absent in Group I rats, while these features were present in Groups II and III rats. The hyaline deposit was also significantly different in all groups. Mesangial proliferation was focal in Group II and diffuse in Group III, while none of the rats in Group I had mesangial proliferation. Similarly, mesangial cellularity was increased in Groups II and III, while none of the rats in Group I had increased cellularity (Table 1).

**Table 1:** Renal histopathology in terms of glomeruli Significance level*: p-value < 0.05 (Chi-square test)

Parameters		Groups			p-value*
		Group I	Group II	Group III	
Magnesium	Expanded	0 (0%)	10 (100%)	10 (100%)	0.000
	Normal	10 (100%)	0 (0%)	0 (0%)	
Capillary wall	Thickened	0 (0%)	10 (100%)	10 (100%)	0.000
	Normal	10 (100%)	0 (0%)	0 (0%)	
Necrosis	Present	0 (0%)	10 (100%)	10 (100%)	0.000
	Absent	10 (100%)	0 (0%)	0 (0%)	
Thrombosis	Present	0 (0%)	10 (100%)	10 (100%)	0.000
	Absent	10 (100%)	0 (0%)	0 (0%)	
Deposits (fibrin)	Present	0 (0%)	10 (100%)	5 (50%)	0.000
	Absent	10 (100%)	0 (0%)	5 (50%)	
Crescent formation	Present	0 (0%)	10 (100%)	5 (50%)	0.000
	Absent	10 (100%)	0 (0%)	5 (50%)	
Sclerosis	Present	0 (0%)	10 (100%)	8 (80%)	0.000
	Absent	10 (100%)	0 (0%)	2 (20%)	
Hyaline deposits	Focal	10 (100%)	0 (0%)	0 (0%)	0.000
	Diffuse	0 (0%)	10 (100%)	10 (100%)	

Renal tubules

Renal histopathology in terms of tubules showed that epithelial necrosis and dilatation were significantly different in all groups. None of the rats in Group I had epithelial necrosis and dilatation. The basement membrane was normal in Group I rats, while the Group II rats all had thickened basement membrane, and in Group III, seven rats had thickened basement membrane. Vacuolization was also significantly different in all groups (Table 2).

**Table 2:** Renal histopathology in terms of tubules Significance level*: p-value < 0.05 (Chi-square test)

Parameters		Groups			p-value*
		Group I	Group II	Group III	
Epithelial necrosis	Present	0 (0%)	10 (100%)	10 (100%)	0.000
	Absent	10 (100%)	0 (0%)	0 (0%)	
Dilatation	Present	0 (0%)	10 (100%)	10 (100%)	0.097
	Absent	10 (100%)	0 (0%)	0 (0%)	
Casts	RBCs	0 (0%)	10 (100%)	10 (100%)	0.000
	WBCs	0 (0%)	10 (100%)	0 (0%)	
	Proteins	0 (0%)	10 (100%)	10 (100%)	
Crystals	Present	0 (0%)	0 (0%)	0 (0%)	-
	Absent	10 (100%)	10 (100%)	10 (100%)	
Basement membrane	Normal	10 (100%)	0 (0%)	0 (0%)	0.000
	Thickened	0 (0%)	10 (100%)	7 (70%)	
	Thickened + focally thick	0 (0%)	0 (0%)	3 (30%)	
Vacuolization	Present	0 (0%)	10 (100%)	10 (100%)	0.000
	Absent	10 (100%)	0 (0%)	0 (0%)	

Interstitium

**Figure 1:**
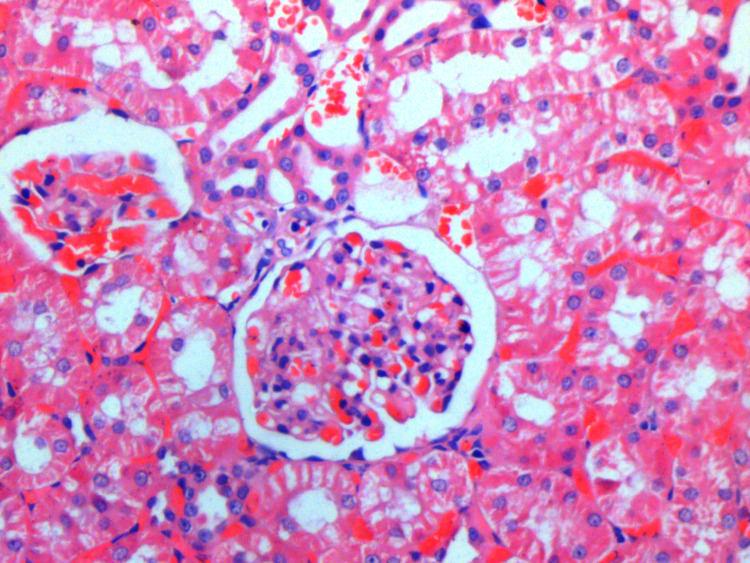
Renal histopathology of Group I (control) showing normal morphology (H&E: 20×)

**Figure 2:**
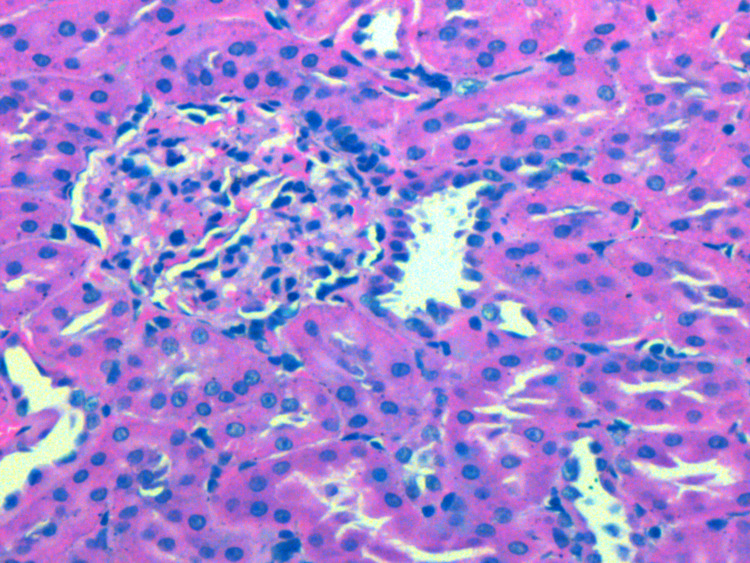
Renal histopathology of Group II showing interstitial edema and inflammation

**Figure 3:**
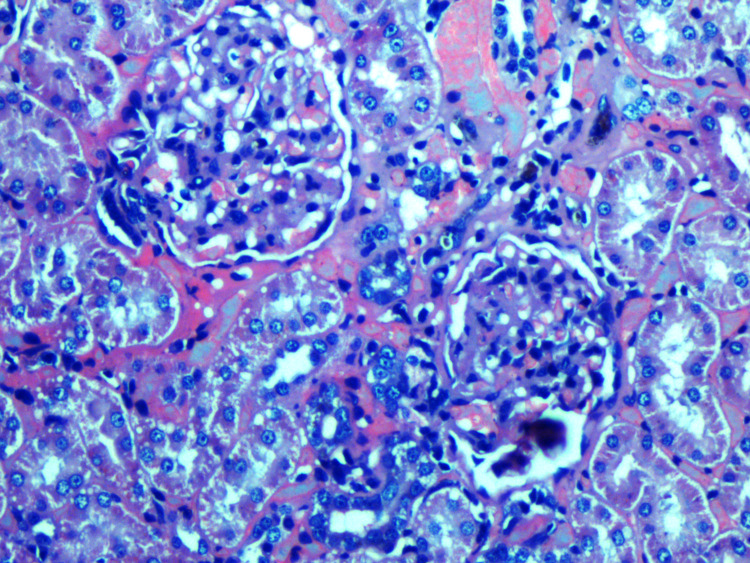
Renal histopathology of Group III showing marked inflammation

Renal histopathology in terms of interstitium showed that edema, inflammation, and fibrosis were significantly different in all groups. In Group I, none of the rats had edema, inflammation, and fibrosis, while in Groups II and III, these characteristics were observed (Table 3, Figures 1, 2, 3).

**Table 3:** Renal histopathology in terms of the interstitium Significance level*: p-value < 0.05 (Chi-square test)

Parameters		Groups			p-value*
		Group I	Group II	Group III	
Edema	Present	0 (0%)	10 (100%)	10 (100%)	0.000
	Absent	10 (100%)	0 (0%)	0 (0%)	
Inflammation	Present	0 (0%)	10 (100%)	10 (100%)	0.000
	Absent	10 (100%)	0 (0%)	0 (0%)	
Fibrosis	Present	0 (0%)	10 (100%)	8 (80%)	0.000
	Absent	10 (100%)	0 (0%)	2 (20%)	

## Discussion

Copper is an obligatory heavy metal that is toxic to humans and animals when acutely or chronically ingested in excessive amounts [14].

Several studies have reported heavy metal toxicity. The oral cavity, digestive system, kidneys, and liver are the delicate targets of toxicity of copper heavy metal. This study confirms that heavy metal toxicity in the kidneys manifests as edema, prolonged inflammation, and fibrosis [15].

Many studies have elucidated that copper toxicity leads to irreversible renal morphological changes [16]. In 2015, deranged renal parameters as a result of renal damage were observed in rats, which was also shown in our study.

The degree of inflammation of the interstitial system in this study was similar to those in other studies. In addition, the current study shows that the degree of renal tissue damage is dose-dependent. Furthermore, the renal histological changes due to copper nephrotoxicity are similar to those of previous studies that reported on the effects of the absorption of excessive amounts of copper [17]. Heavy metals induce and stimulate several mechanisms that subsequently cause changes in the blood vessels and inflammation [18]. Moreover, researchers have observed the effects of copper toxicity on the profile of lipid compounds and tissue damage from the resulting oxidative stress [19].

In one study, researchers studied the effects of copper on the kidneys and liver of 18 rats and found that copper toxicity resulted in irreversible tissue damage in both organs [18]. Another study involving four groups of rats reported that the renal parenchyma exhibited changes consistent with copper toxicity four weeks after copper deposition in renal tissues [19]. In the present study, the presence of interstitial fibrosis was also observed in Group I and Groups II and III, respectively.

Our study revealed that copper induces renal toxicity in a dose-dependent manner. There are a few limitations of this study. Firstly, we only considered the toxic effects of copper on histological changes in the kidneys as the outcome variable, although these effects should also be investigated on other vital organs such as the liver of rodents. Secondly, other parameters such as lipid profile, protein profile, and kidney functions (creatinine, urea, and uric acid) were ignored, which are however important and must be considered. Further studies are necessary to determine the association between renal toxicity and copper ingestion.

## Conclusions

Copper is recommended in the form of different herbs (that contain copper) by traditional health practitioners (Hakeems) and Quakes for the treatment of various chronic ailments in our population. However, prolonged use can result in irreversible damage to the kidneys and other vital organs of the body. Further studies should be undertaken to determine the health hazards of copper in the local population as such types of practices are very common in Pakistan.
